# Alcalase-hydrolyzed casein peptides after simulated gastrointestinal digestion alleviate high-fat diet-induced metabolic dysfunction in mice

**DOI:** 10.3389/fnut.2026.1960914

**Published:** 2026-09-15

**Authors:** Ling Mo, Chaochao Zhao, Tian Wang, Wanli Xu, Yingping Deng, Yongming Yu, Xiaoyun Zeng

**Affiliations:** 1 Wuzhou Red Cross Hospital, Wuzhou, Guangxi, China; 2 School of Public Health, Guilin Medical University, Guilin, Guangxi, China; 3 Guangxi Key Laboratory of Diabetic Systems Medicine, Guilin Medical University, Guilin, China

**Keywords:** gut microbiota, hypolipidemic peptides, metabolomics, milk protein, simulated gastrointestinal digestion

## Abstract

**Background and aim:**

High-fat diet (HFD)-induced metabolic dysfunction is associated with disturbances in gut microbial composition and hepatic lipid metabolism. This study investigated the metabolic effects of Alcalase-hydrolyzed casein peptides after simulated gastrointestinal digestion (MCPs) in HFD-fed mice and explored their potential association with gut microbiota and fecal metabolic alterations.

**Results:**

MCP supplementation attenuated body weight gain, improved serum lipid profiles, and alleviated hepatic steatosis and injury in HFD-fed mice. In the colon, MCPs ameliorated histological damage and suppressed the mRNA and protein expression of pro-inflammatory cytokines, including IL-6, IL-1β, and TNF-α. Gut microbiota analysis revealed increased relative abundances of *Akkermansia, Bifidobacterium*, and *Lactobacillus* and reduced abundance of *Desulfovibrio* following MCP supplementation. Fecal metabolomics identified alterations in bile acid- and lipid metabolism-related signatures, including increased levels of tauroursodeoxycholic acid (TUDCA) and reduced arachidonic acid metabolism-related metabolites. In the liver, MCPs downregulated. SREBP-1c/ACC/GPAT1 lipogenic signaling and enhanced Nrf2/HO-1-associated antioxidant responses. Correlation analysis revealed associations between microbial-metabolic alterations and host metabolic phenotypes.

**Conclusion:**

Collectively, MCPs may alleviate HFD-induced metabolic dysfunction by modulating gut microbiota-associated metabolic profiles and improving intestinal and hepatic metabolic responses.

## Graphical abstract

**Figure:**
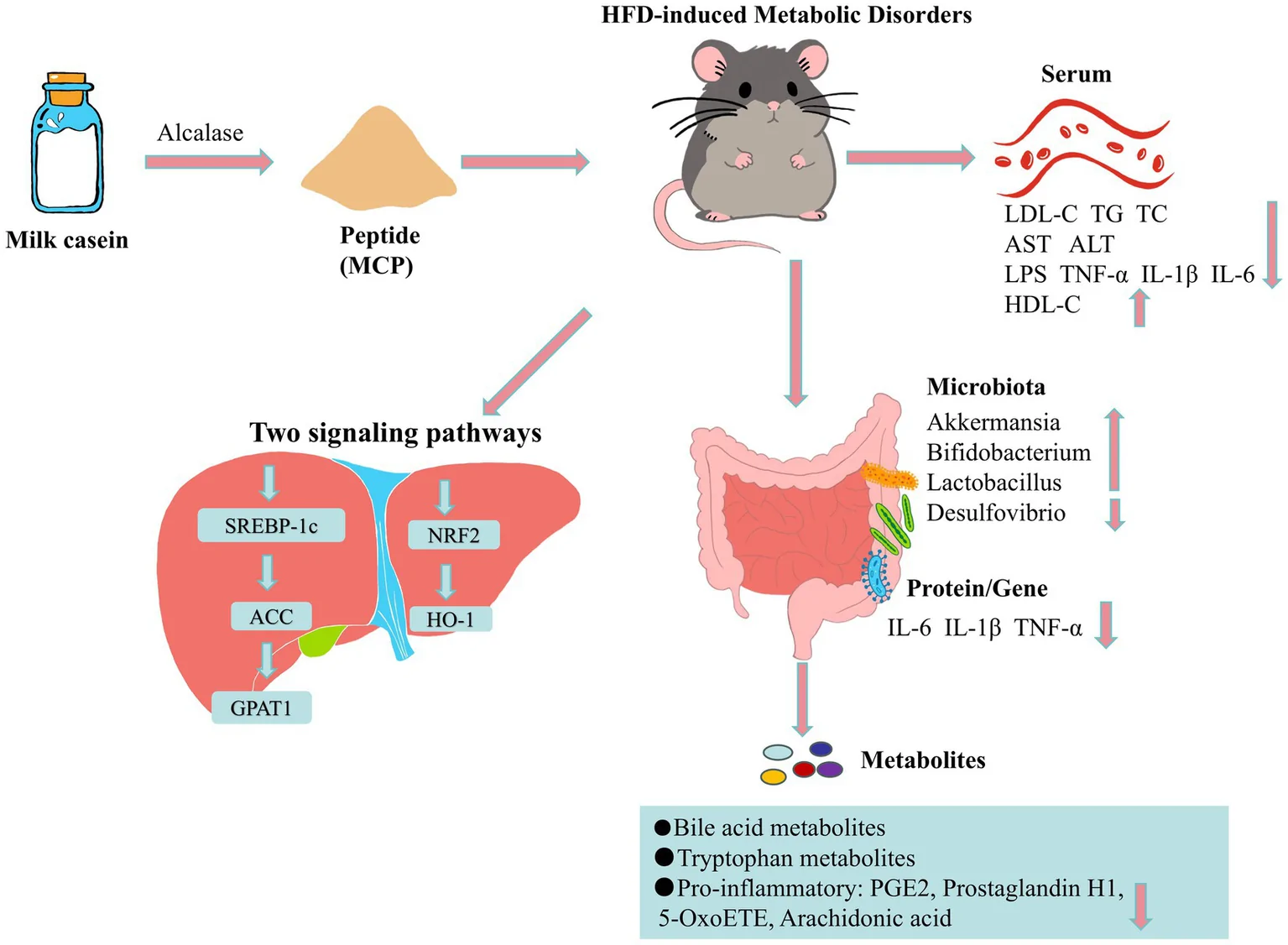

## 1 Introduction

Hyperlipidemia is a major metabolic abnormality associated with diet-related metabolic dysfunction and contributes to metabolic and cardiovascular complications, including non-alcoholic fatty liver disease and atherosclerotic cardiovascular disease (1, 2). Long-term consumption of a high-fat diet (HFD) disrupts lipid homeostasis and promotes chronic low-grade inflammation (3, 4). HFD-induced gut microbial dysbiosis and impaired intestinal barrier function may facilitate lipopolysaccharide (LPS) translocation into the circulation, contributing to metabolic endotoxemia (5). Circulating LPS can activate Toll-like receptor 4 (TLR4)/NF-κB signaling in hepatic and adipose tissues and aggravate metabolic inflammation (6). These inflammatory responses are closely associated with hepatic oxidative stress, dysregulated lipogenic signaling, and excessive lipid accumulation (7, 8). Therefore, dietary strategies that simultaneously improve intestinal homeostasis and hepatic lipid metabolism may offer an effective approach to alleviating HFD-induced metabolic dysfunction.

The gut microbiota is a key mediator linking dietary exposure to intestinal and hepatic metabolic homeostasis. HFD consumption alters microbial community structure, reducing beneficial taxa associated with intestinal homeostasis and enriching potentially pro-inflammatory microorganisms (9–11). Such alterations may impair mucosal barrier function and disturb host energy and lipid metabolism (12–14). Beyond barrier regulation, the gut microbiota also influences host physiology through the production and transformation of small-molecule metabolites. Untargeted metabolomics studies have identified host microbial co-metabolic alterations that link the intestinal luminal environment with hepatic function (15, 16). Bile acid metabolism is a critical component of this host–microbial interaction. Microbial bile salt hydrolases deconjugate primary bile acids, which subsequently undergo further microbial transformation into secondary bile acids (17). Tauroursodeoxycholic acid (TUDCA), a taurine-conjugated derivative of ursodeoxycholic acid, exerts potent cytoprotective and anti-inflammatory effects (18). Conversely, dysregulated arachidonic acid metabolism may promote inflammatory lipid signaling and contribute to intestinal and hepatic injury (19). Joint characterization of the gut microbiota and fecal metabolome may therefore provide valuable insights into microbial-metabolic alterations associated with HFD-induced gut-liver dysfunction.

Dietary intervention is considered an effective approach for managing hyperlipidemia. Food-derived bioactive peptides have attracted increasing attention as potential nutritional ingredients because of their diverse biological activities and favorable safety profiles. These peptides can be generated from dietary proteins through enzymatic hydrolysis or fermentation processes (20–22). Casein, the major protein fraction in bovine milk, contains encrypted peptide sequences that can be generated through enzymatic hydrolysis and may contribute to metabolic regulation. Enzymatic hydrolysis using food-grade proteases represents an effective strategy to generate casein-derived peptide mixtures with potential biological activities (23). Alcalase is frequently utilized because of its broad substrate specificity and efficient peptide-generating capacity. Although previous studies have shown that conventional casein-derived peptides may exert antioxidant, anti-inflammatory, and metabolic regulatory activities in cell and animal models (24, 25), many of these studies have overlooked the profound impact of physiological digestion. Following oral consumption, casein-derived peptides are exposed to gastrointestinal proteolysis, which may modify their peptide profiles and influence their structural characteristics and biological activities. Simulated gastrointestinal digestion is therefore commonly used to evaluate whether food-derived peptide preparations retain structural stability and potential biological activity under gastrointestinal conditions (26). Peptide fractions that withstand gastrointestinal processing may retain biological activity and represent promising candidates for evaluating food-derived functional ingredients. However, whether casein-derived peptides after simulated gastrointestinal digestion can alleviate HFD-induced metabolic dysfunction and whether these effects are associated with gut microbiota and fecal metabolic alterations remain insufficiently understood.

In this study, a casein peptide preparation obtained through alcalase hydrolysis followed by simulated gastrointestinal digestion was used for animal intervention. The resulting alcalase-hydrolyzed casein peptides after simulated gastrointestinal digestion (MCPs) were administered to HFD-fed mice to evaluate their effects on systemic lipid metabolism, colonic inflammation, hepatic steatosis, and oxidative stress. In addition, 16S rRNA gene sequencing and untargeted fecal metabolomics were employed to characterize MCP-associated alterations in the gut microbial and metabolic profiles and their associations with intestinal and hepatic phenotypes. The findings may provide insights into the functional potential of digestion-processed casein peptides and support their application as dairy-derived functional ingredients.

## 2 Materials and methods

### 2.1 Chemicals

Alcalase, pepsin and trypsin were obtained from Shanghai Yuanye Biotechnology Co., Ltd. (Shanghai, China). Pre-stained protein marker was supplied by Beijing Prolux Biotechnology Co., Ltd. (Beijing, China). Commercial assay kits for total cholesterol (TC), triglycerides (TG), low-density lipoprotein cholesterol (LDL-C), high-density lipoprotein cholesterol (HDL-C), aspartate aminotransferase (AST), alanine aminotransferase (ALT), total superoxide dismutase (T-SOD), catalase (CAT), glutathione peroxidase (GSH-Px), and malondialdehyde (MDA) were purchased from Nanjing Jiancheng Bioengineering Institute (Nanjing, China). Mouse-specific enzyme-linked immunosorbent assay (ELISA) kits for interleukin-1β (IL-1β), interleukin-6 (IL-6), and tumor necrosis factor-α (TNF-α) were obtained from Nanjing Jiancheng Biotechnology Co., Ltd. The lipopolysaccharide (LPS) kit was purchased from Huamei Biological Engineering Co., Ltd. (Wuhan, China). The BCA protein assay kit was obtained from Shanghai Beyotime Biotechnology Co., Ltd. (Shanghai, China). TRIzol reagent, the first-strand cDNA synthesis kit, and the SYBR Green qPCR master mix were purchased from Thermo Fisher Scientific (Waltham, MA, United States). Primary antibodies against GAPDH, IL-1β, IL-6, TNF-α, and SREBP-1c were sourced from Proteintech (Wuhan, China), while antibodies against Nrf2, ACC, and GPAT1 were provided by Affinity Biosciences (Jiangsu, China). The secondary antibody, HRP-conjugated AffiniPure Goat Anti-Rabbit IgG, was also purchased from Proteintech.

### 2.2 Preparation and simulated gastrointestinal digestion of milk casein peptides

The milk casein peptides (MCPs) were prepared with reference to the previously described method (27). Casein was dispersed in distilled water at 5% (w/v), and the suspension was adjusted to pH 8.0. Alcalase was added at an enzyme-to-substrate ratio of 2%, and hydrolysis was conducted at 55 °C for 4 h. The pH was maintained at 8.0 by adding 1 mol/L NaOH during hydrolysis. Alcalase was subsequently inactivated by heating the hydrolysate at 95 °C for 10 min. The hydrolysate was cooled to room temperature and centrifuged at 4000 × g for 20 min. The supernatant was collected and subjected to simulated gastrointestinal digestion using an adapted INFOGEST static digestion procedure. Because the casein hydrolysate contained negligible starch, salivary α-amylase was omitted. Briefly, for the gastric phase, simulated gastric fluid (SGF) containing pepsin (final activity of 2000 U/mL) was added to the supernatant. The mixture was adjusted to pH 3.0 using 1 mol/L HCl and incubated at 37 °C for 2 h under continuous shaking. Subsequently, for the intestinal phase, simulated intestinal fluid (SIF) containing pancreatin (final activity of 100 U/mL based on trypsin activity) and bile salts (10 mmol/L) was added to the gastric digest. The mixture was adjusted to pH 7.0 using 1 mol/L NaOH and incubated at 37 °C for another 2 h. To terminate the digestion process, the gastrointestinal digest was rapidly heated at 95 °C for 10 min to inactivate the digestive enzymes, then cooled on ice and centrifuged at 10000 × g for 20 min at 4 °C. The supernatant was collected, freeze-dried, and designated as milk casein peptides (MCPs). The final lyophilized MCP powder was stored at −20 °C until animal administration.

### 2.3 Animals and experimental design

Animal experiments were approved by the Institutional Animal Care and Use Committee of Guilin Medical University (Animal Experiment License Number: SCXK (Yue) 2022-0063; Ethical Certificate Number: GLMU-IACUC-202310409). Healthy male C57BL/6 J mice (aged 6 weeks) were obtained from Guangdong Vitron Life Science Technology Co., Ltd. (Guangdong, China). All mice were housed in a specific pathogen-free (SPF) environment (temperature 24 ± 1 °C, relative humidity 55 ± 5%, light/dark cycle 12 h) and acclimatized for 1 week. The normal chow diet (NCD, D12450J, 10% kcal from fat) and high-fat diet (HFD, D12492, 60% kcal from fat) were purchased from Jiangsu Synergy Pharmaceutical Bioengineering Co., Ltd. (China).

After acclimatization, the mice were randomly divided into four groups (*n* = 10 per group) based on initial body weights: (1) ND group (fed a normal diet and administered saline via oral gavage); (2) ND + P group (fed a normal diet and administered MCP (600 mg/kg) via oral gavage); (3) HFD group (fed an HFD and administered saline via oral gavage); and (4) HFD + P group (fed an HFD and administered MCP (600 mg/kg) via oral gavage). The intervention period lasted for exactly 12 weeks. During this period, saline or MCPs were administered once daily. Body weight and food intake were recorded weekly, and the actual gavage volume for each mouse was dynamically adjusted according to their most recently recorded body weight. The experimental procedure is shown in Figure 1A.

**Figure 1:**
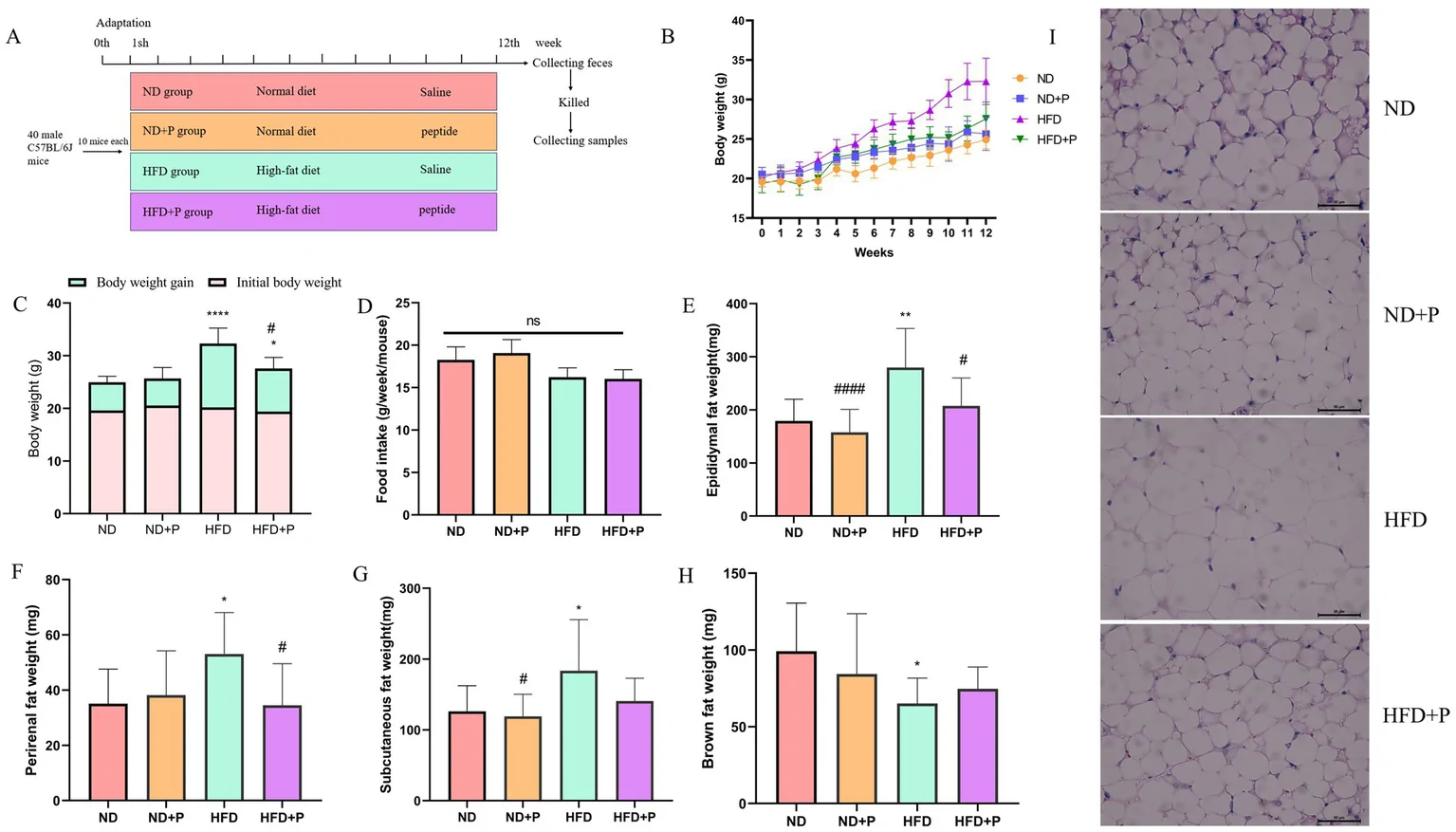
Effects of MCP intervention on body weight and adiposity in mice fed a high-fat diet (HFD). **(A)** Experimental procedure. **(B)** Changes in mouse body weight during the 12-week intervention. **(C)** Final body weight. **(D)** Food intake. **(E)** Subcutaneous fat weight. **(F)** Perirenal fat weight. **(G)** Epididymal fat weight. **(H)** Brown fat weight. **(I)** Representative H&E-stained sections of perirenal adipose tissue. ^*^ The groups compared with ND group, ^#^ the groups compared with HFD group. Analyzed by Tukey test and one-way ANOVA. ^*^: *p* < 0.05; ^**^: *p* < 0.01; ^***^: *p* < 0.001; ^****^: *p* < 0.0001; ^#^: *p* < 0.05; ^##^: *p* < 0.01; ^###^: *p* < 0.001; ^####^: *p* < 0.0001.

During the final week of the experiment, fresh feces were collected, snap-frozen in liquid nitrogen, and stored at −80 °C for subsequent microbiota and metabolomics analyses. At the end of the 12-week intervention period, following a 12-h overnight fast, all mice were anesthetized with narcolan. Blood was drawn from the eyeballs, and serum was separated by centrifugation. Liver and epididymal fat tissues were quickly dissected, rinsed with ice-cold 0.9% saline, weighed and either fixed in 4% paraformaldehyde or stored at −80 °C for further analysis. The organ index was calculated as: (tissue weight / body weight) × 100%.

### 2.4 Serum and tissue biochemical analyses

Centrifugation (4 °C, 3500 × g, 15 min) was used to separate serum from the blood. The levels of serum total cholesterol (TC), triglycerides (TG), low-density lipoprotein cholesterol (LDL-C), high-density lipoprotein cholesterol (HDL-C), aspartate aminotransferase (AST), alanine aminotransferase (ALT) and ‌Lipopolysaccharide‌ (LPS) were measured according to the manufacturer’s instructions.

For hepatic biochemical parameters, liver tissue samples were homogenized in ice-cold 0.9% saline (1:9, w/v) and centrifuged at 4000 × g for 15 min. The supernatants were collected to determine liver TC, TG, superoxide dismutase (SOD), malondialdehyde (MDA), catalase (CAT), and glutathione peroxidase (GSH-Px) contents using respective commercial kits. The levels of hepatic inflammatory cytokines, including tumor necrosis factor-alpha (TNF-α), interleukin-1beta (IL-1β), and interleukin-6 (IL-6), were quantified using mouse-specific ELISA kits.

### 2.5 Histopathological evaluation

The liver, epididymal adipose tissues, jejunum, and colon were fixed in 4% paraformaldehyde for 24 h and embedded in paraffin. The tissue blocks were sectioned to a thickness of approximately 5 μm (Thermo Histostar, United States). After dewaxing and rehydration, the sections were stained with hematoxylin and eosin (H&E) to evaluate lipid accumulation in the liver and adipose tissues, as well as morphological changes in the jejunum and colon. The stained sections were examined and photographed under a light microscope (Leica, Solms, Germany) at 400 × magnification. The average adipocyte area in the epididymal fat was quantified using Image-Pro Plus 6.0 software (Media Cybernetics, United States).

### 2.6 Sequencing analysis of intestinal microbial diversity

Total genomic DNA was extracted from the fecal samples using the OMEGA Soil DNA Kit (Omega Bio-Tek, United States). The quantity and quality of DNA were assessed via a NanoDrop ND-1000 spectrophotometer and agarose gel electrophoresis. The V3–V4 region of the bacterial 16S rRNA gene was amplified using the forward primer 338F (5′-ACTCCTACGGGAGGCAGCAG-3′) and the reverse primer 806R (5′-GGACTACHVGGGTWTCTAAT-3′). Amplicon sequencing was performed on the Illumina MiSeq platform by Shanghai Personal Biotechnology Co., Ltd. Sequence data and microbiome bioinformatics (including alpha-diversity and PCoA for beta-diversity) were processed using QIIME2 and the R package (v3.2.0).

### 2.7 Fecal metabolomics based on UHPLC-QTOF MS

The feces of each mouse in the four groups were collected in sterile tubes for the following metabolomic analysis. The UHPLC-Q-TOF MS-based metabolomics analysis was performed by Shanghai Personal Biotechnology Co., Ltd. (Shanghai, China), using an Agilent 1,290 infinity LC ultrahigh-performance liquid chromatography system coupled with an AB triple TOF 6600 mass spectrometer. Metabolite identification was achieved by matching accurate mass and MS/MS spectra against authoritative public databases, including HMDB and KEGG. After normalization to the total peak intensity, the processed data were analyzed using the R package (ropls), where they were subjected to multivariate data analysis, including Pareto-scaled principal component analysis (PCA) and orthogonal partial least squares discriminant analysis (OPLS-DA). Seven-fold cross-validation and response permutation testing were used to evaluate the robustness of the model. For differential feature selection, the variable importance in the projection (VIP) value of each variable in the OPLS-DA model was calculated to indicate its contribution to the classification. Metabolites with VIP values >1 were further subjected to Student’s *t* test at the univariate level to measure the significance of each metabolite, and *P* values less than 0.05 were considered statistically significant.

### 2.8 RNA extraction and qRT-PCR detection

Liver or colon tissue (0.1 g) was collected from each group of mice for total RNA extraction according to the manufacturer’s protocol. Specifically, colonic tissues were exclusively used for evaluating the expression of inflammatory cytokines. The Novoscript Plus all-in-one first-strand cDNA synthesis Supermix (gDNA pure) kit was used to synthesize cDNA. The target gene primers were designed using the gene sequence information provided by the NCBI database and Primer Premier 5.0 software, and synthesized by General Biol Company, China. The NovoStart SYBR qPCR Supermix plus kit was used for quantitative real-time reverse transcription polymerase chain reaction (qRT-PCR) in a LightCycler 96 real-time PCR system (Roche Diagnostics) in triplicate. Gapdh was used as an endogenous control to normalize the expression levels of the target gene mRNA.

### 2.9 Western blot analysis

RIPA buffer (Beyotime, China) supplemented with protease and phosphatase inhibitor cocktails (Abiowell, China) was used to extract total protein from mouse liver tissues. The homogenates were centrifuged at 4 °C, 12000 × g for 15 min to remove the precipitate. The protein concentration of the supernatant was determined using a BCA Protein Assay kit. Equal amounts of extracted protein were separated by 10% (wt/vol) SDS-PAGE and transferred to an NC membrane (Abiowell, China). The membranes were blocked for 2 h with PBST containing 5% skimmed milk at room temperature and then incubated with primary antibodies overnight at 4 °C. Subsequently, the membranes were then washed three times for 15 min with PBST and incubated with horseradish peroxidase (HRP)-conjugated secondary antibody for 85 min at room temperature. Finally, they were washed three times with PBST for 15 min each time, incubated with ECL Plus reagent (Abiowell, China) for 5 min, and visualized using a Bio-Rad Laboratories imaging system.

### 2.10 Statistical and bioinformatics analysis

Data are presented as the means ± standard deviations (SD). Statistical analyses were performed using GraphPad Prism 8.0.2. Significant differences among groups were evaluated by one-way analysis of variance (ANOVA) followed by Tukey’s *post hoc* test. A *p*-value <0.05 was considered statistically significant. LEfSe analysis was conducted using an LDA threshold of 2.0 to identify differential bacterial taxa. Spearman correlation coefficients were calculated to reveal associations between differential gut microbiota and key metabolites, with networks visualized using corresponding software (*p* < 0.05).

## 3 Results

### 3.1 MCP attenuates HFD-induced obesity

The experimental design and intervention schedule are illustrated in Figure 1A. Body weight changes were monitored throughout the intervention period to evaluate the effects of MCP supplementation on HFD-induced weight gain. An overall upward trend in body weight was observed across all groups (Figure 1B). The body weight curve of the HFD group began to diverge from that of the ND group from the fifth week onward. By the end of the 12-week intervention, HFD feeding resulted in a significant increase of 24.59% in final body weight compared with the ND group. Conversely, MCP intake effectively attenuated body weight gain (Figure 1C). Furthermore, HFD feeding significantly increased perirenal fat, epididymal fat, and subcutaneous fat weights in the HFD group compared to the ND group, alongside a notable decrease in brown fat weight (Figures 1E–H; *p* < 0.05). Notably, MCP supplementation markedly reduced adipose tissue accumulation induced by HFD feeding; the weights of these white fat pads, epididymal fat in the HFD + P group decreased significantly relative to the HFD group (*p* < 0.05). Throughout the intervention period, food intake did not differ significantly among the high-fat diet-fed groups (Figure 1D), suggesting that MCP-mediated metabolic improvements were unlikely to be explained solely by reduced food intake. Additionally, morphological alterations in epididymal adipocytes were evaluated via H&E staining. Individual adipocytes in the HFD group were substantially enlarged, whereas MCP supplementation significantly inhibited this adipocyte hypertrophy (Figure 1I). These findings indicate that MCP supplementation attenuated HFD-induced adipose expansion without affecting food intake.

### 3.2 MCP ameliorates dyslipidemia

To investigate the impact of MCP on lipid disorders and liver function, we analyzed serum levels of total cholesterol (TC), triglycerides (TG), low-density lipoprotein cholesterol (LDL-C), and high-density lipoprotein cholesterol (HDL-C). HFD feeding significantly increased serum TC, TG, and LDL-C levels while decreasing HDL-C levels (Figures 2A–D). Compared with the HFD group, MCP supplementation reduced serum TG, TC, and LDL-C levels by 27.26, 48.89, and 27.36%, respectively, while increasing HDL-C levels by 30.16%. Additionally, we assessed liver damage by measuring serum ALT and AST activities, which are classic biomarkers for hepatocyte injury. The HFD group displayed a significant 2.24-fold increase in ALT and a 24.07% increase in AST levels compared to the ND group (Figures 2E–F). Following MCP intervention, these transaminase activities were notably restored to near-normal levels (Figures 2E–F). Moreover, analysis of serum inflammatory markers demonstrated significantly higher TNF-α, IL-6, and IL-1β concentrations in the HFD group, indicating systemic inflammation triggered by the high-fat diet. However, after MCP treatment, the secretion of these pro-inflammatory cytokines was significantly decreased (Figures 2H–J), accompanied by a simultaneous decline in serum lipopolysaccharide (LPS) levels (Figure 2G).

**Figure 2:**
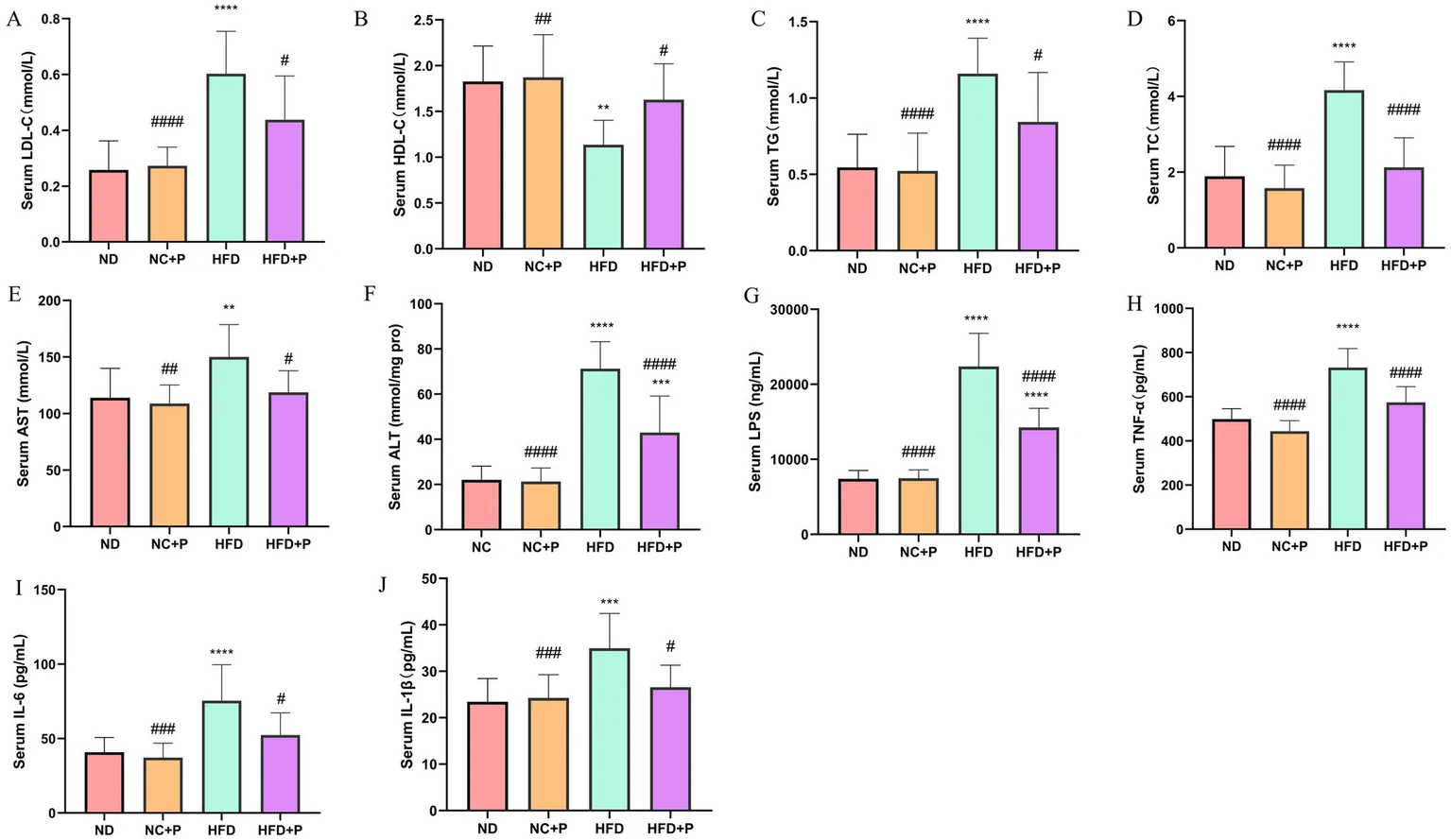
Effects of MCP intervention on serum lipid profiles and circulating inflammatory markers in HFD-fed mice. **(A)** Serum LDL-C, **(B)** Serum HDL-C, **(C)** Serum TG, **(D)** Serum TC, **(E)** Serum AST, **(F)** Serum ALT, **(G)** Serum LPS, **(H)** Serum TNF-α, **(I)** Serum IL-6, **(J)** Serum IL-1β. ^*^ The groups compared with ND group, ^#^ the groups compared with HFD group. Analyzed by Tukey test and one-way ANOVA. ^*^: *p* < 0.05; ^**^: *p* < 0.01; ^***^: *p* < 0.001; ^****^: *p* < 0.0001; ^#^: *p* < 0.05; ^##^: *p* < 0.01; ^###^: *p* < 0.001; ^####^: *p* < 0.0001.

### 3.3 MCP alleviates hepatic steatosis

A high-fat diet typically induces hepatic steatosis, inflammation, and structural abnormalities. Representative anatomical images demonstrate that the liver enlargement and visceral fat accumulation in mice from the HFD + P group were significantly reduced (Figure 3A). Furthermore, the significant elevation of liver tissue weight in the HFD group was effectively reversed following MCP intervention (Figure 3E). To assess the effects of MCP on lipid deposition, we further examined the histopathological characteristics of liver tissues. As shown in Figure 3B, numerous hepatocytes in the HFD group exhibited severe steatosis, with lipid droplets of varying sizes apparent in the cytoplasm. Many hepatocytes displayed edema, loose cytoplasm, and cell swelling, while a subset showed hepatocyte ballooning, cytoplasmic vacuolation, and inflammatory infiltration. Following MCP intervention, the liver tissue showed significant improvement, with a marked reduction in both lipid droplet accumulation and inflammatory lesions. These histological improvements confirm that MCP ameliorates HFD-induced hepatic steatosis, consistent with the observed changes in liver weight.

**Figure 3:**
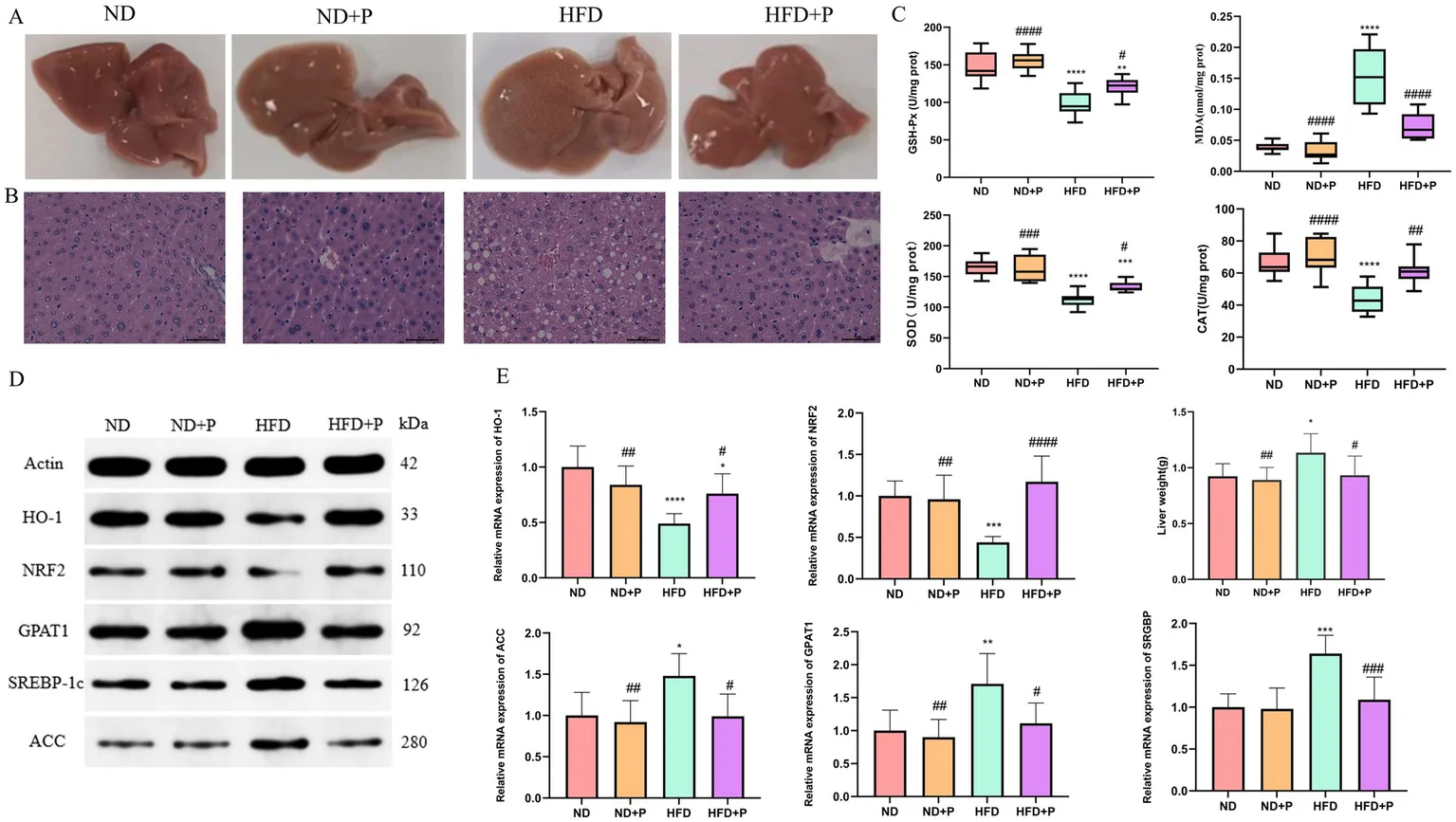
Effects of MCP intervention on liver steatosis and function in HFD-fed mice. **(A)** Gross liver morphology of mice in each group. **(B)** Representative H&E-stained liver sections. **(C)** Hepatic oxidative stress indicators (GSH-Px, MDA, SOD, CAT). **(D)** Representative Western blot images of proteins related to lipogenesis and antioxidant capacity. **(E)** Relative mRNA expression levels related to lipogenesis and antioxidant capacity, and liver weight. ^*^ The groups compared with ND group, ^#^ the groups compared with HFD group. Analyzed by Tukey test and one-way ANOVA. ^*^: *p* < 0.05; ^**^: *p* < 0.01; ^***^: *p* < 0.001; ^****^: *p* < 0.0001; ^#^: *p* < 0.05; ^##^: *p* < 0.01; ^###^: *p* < 0.001; ^####^: *p* < 0.0001.

### 3.4 MCP improves intestinal morphology and inflammation

Histological examination revealed significant structural alterations in the jejunum and colon of HFD-fed mice (Figure 4A). Conversely, intervention with MCP partially preserved intestinal structural integrity. Furthermore, we assessed the levels of pro-inflammatory cytokines in intestinal tissues. Compared to the ND group, both mRNA and protein expressions of TNF-α, IL-1β, and IL-6 were notably elevated in the HFD group (Figures 4B,C). MCP administration significantly downregulated the expression of these pro-inflammatory factors, indicating a protective impact against high-fat diet-induced intestinal inflammation.

**Figure 4:**
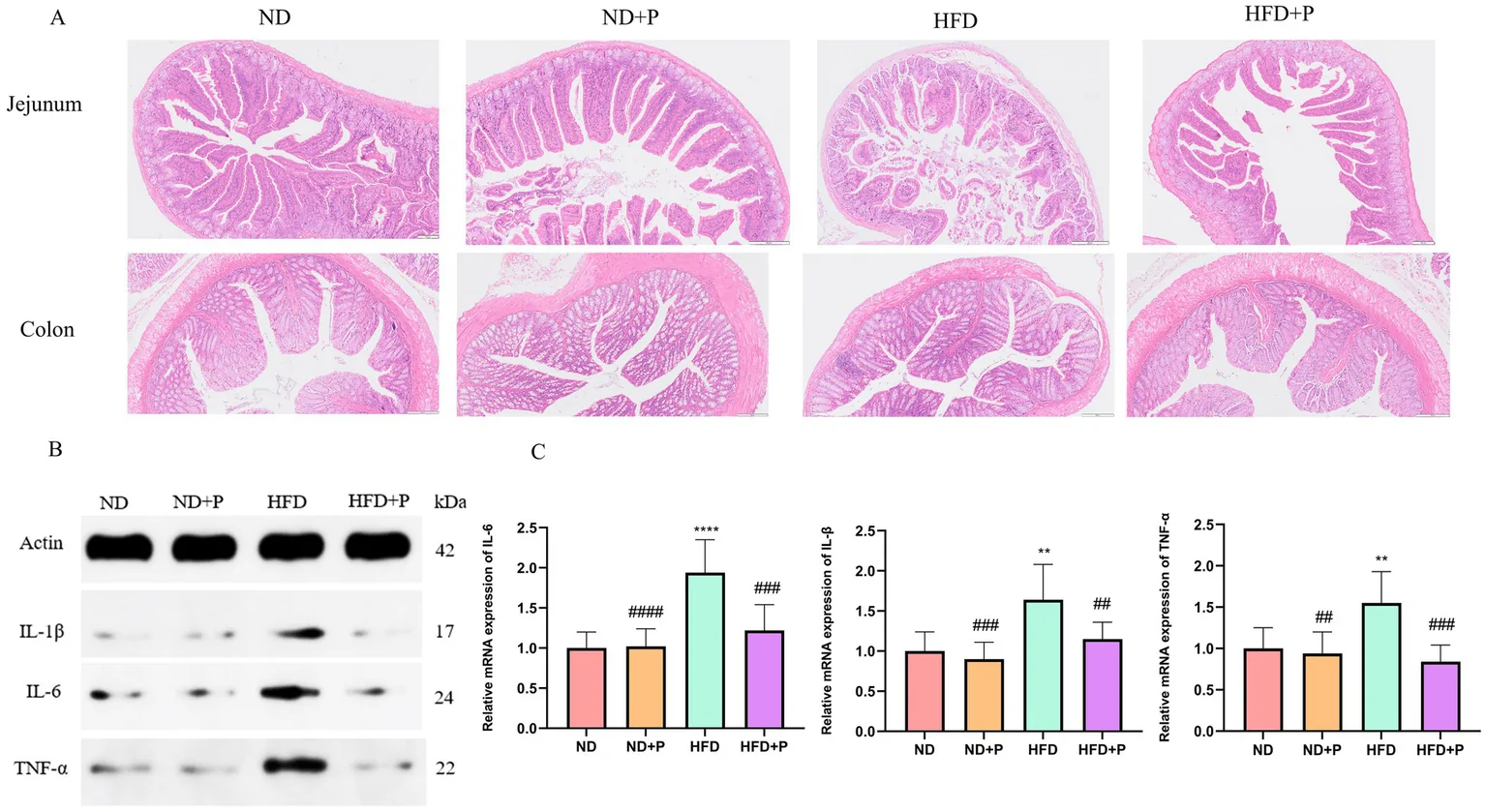
Effects of MCP intervention on intestinal morphology and inflammation in HFD-fed mice. **(A)** Representative H&E-stained sections of the jejunum and colon. **(B)** Representative Western blot images of proteins related to colonic inflammation. **(C)** Relative mRNA expression levels related to colonic inflammation. ^*^ The groups compared with ND group, ^#^ the groups compared with HFD group. Analyzed by Tukey test and one-way ANOVA. ^*^: *p* < 0.05; ^**^: *p* < 0.01; ^***^: *p* < 0.001; ^****^: *p* < 0.0001; ^#^: *p* < 0.05; ^##^: *p* < 0.01; ^###^: *p* < 0.001; ^####^: *p* < 0.0001.

### 3.5 MCP regulates hepatic lipid metabolism

To further investigate the molecular mechanisms underlying MCP-mediated improvement of hepatic metabolism, hepatic lipid metabolism-related proteins and oxidative stress markers were analyzed. Results from Western blotting and RT-qPCR experiments demonstrated that HFD feeding significantly upregulated the protein expression of sterol regulatory element-binding protein 1c (SREBP-1c) and its downstream target enzymes, acetyl-CoA carboxylase (ACC) and glycerol-3-phosphate acyltransferase 1 (GPAT1), indicating hyperactive hepatic lipogenesis (Figures 3D,E). MCP intervention effectively suppressed the SREBP-1c pathway, which was consistent with the improvement in hepatic steatosis observed in histological analyses. Furthermore, because hepatic metabolic overload typically triggers oxidative stress, we assessed the Nrf2/HO-1 antioxidant pathway. We found that MCP treatment significantly increased hepatic Nrf2 expression and upregulated the expression of HO-1, both of which were suppressed in the HFD group (Figures 3D,E). Additionally, MCP effectively ameliorated liver oxidative stress, as evidenced by decreased levels of malondialdehyde (MDA) and restored activities of total superoxide dismutase (T-SOD), catalase (CAT), and glutathione peroxidase (GSH-Px) (Figure 3C). Collectively, these results indicate that MCP mitigates hepatic lipid accumulation and oxidative damage by modulating key lipogenic and antioxidant signaling pathways.

### 3.6 MCP modulates gut microbiota composition

To further investigate the relationship between hyperlipidemia and the gut microbiota, as well as the effects of MCP on microbial regulation, we examined the structure and composition of the fecal microbiota via 16S rRNA gene sequencing. Figure 5B illustrates the unique and common operational taxonomic units (OTUs) in each group, with 184 OTUs being common across all groups. The distribution of unique OTUs in the groups was as follows: 590 in the ND group, 539 in the ND + P group, 273 in the HFD group, and 353 in the HFD + P group, indicating alterations in the gut microbiota of each group. We then analyzed the diversity of the gut microbiota in mice, focusing on α-diversity, which reflects the microbial community’s richness and diversity. The ACE, Chao1, and Sobs index are standard metrics for assessing microbial α-diversity. As depicted in Figures 6B–D, compared to the ND group, the Chao1, Goods_coverage index, Observed_otus, Shannon index, and Simpson indices were reduced in the HFD group, while they were significantly recovered in the HFD + P group, suggesting that MCP intervention could restore gut microbiota diversity (Figures 6A–G). Additionally, principal coordinate analysis (PCoA) was utilized to evaluate the β-diversity among groups, while clustering analysis was conducted to examine the similarities and differences in gut microbiota composition across groups. PCoA1 explained 49.82% of the variance, and PCoA2 accounted for 11.09% (Figure 5A). Notably, the HFD group exhibited distinct clustering separate from the other three groups, whereas a close clustering pattern was observed between the samples of the HFD + P group and the ND group, suggesting that MCP administration normalized the overall microbial community structure.

**Figure 5:**
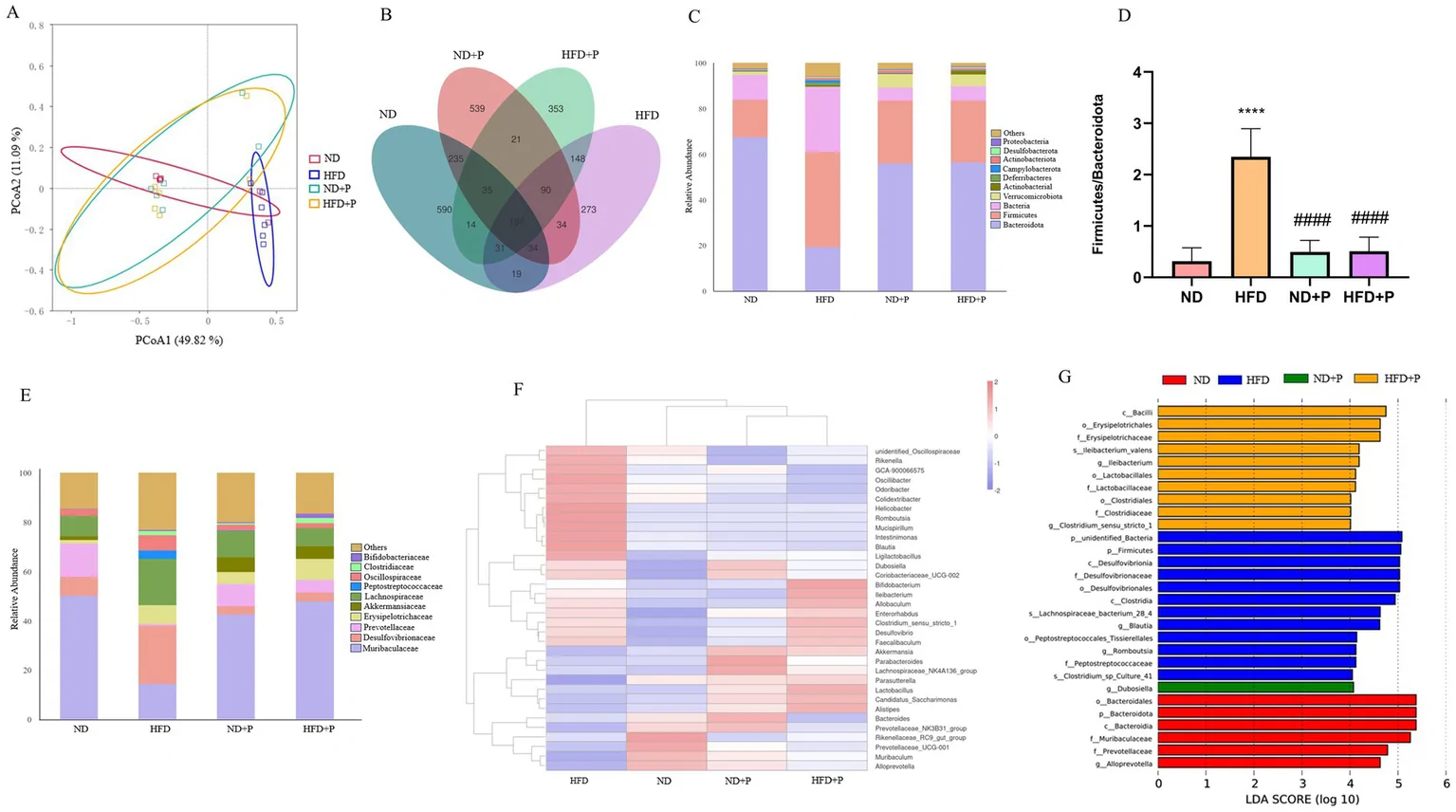
Effects of MCP intervention on the structural composition of fecal microbiota in HFD-fed mice. **(A)** PCoA plot. **(B)** Venn diagram. **(C)** Relative abundance of microbiota at the phylum level. **(D)** Ratio of Firmicutes/Bacteroidota. **(E)** Relative abundance of microbiota at the family level. **(F)** Heatmap showing the relative abundance of microbiota at the genus level. **(G)** LEfSe cladogram/bar chart. ^*^ The groups compared with ND group, ^#^ the groups compared with HFD group. Analyzed by Tukey test and one-way ANOVA. ^*^: *p* < 0.05; ^**^: *p* < 0.01; ^***^: *p* < 0.001; ^****^: *p* < 0.0001; ^#^: *p* < 0.05; ^##^: *p* < 0.01; ^###^: *p* < 0.001; ^####^: *p* < 0.0001.

**Figure 6:**
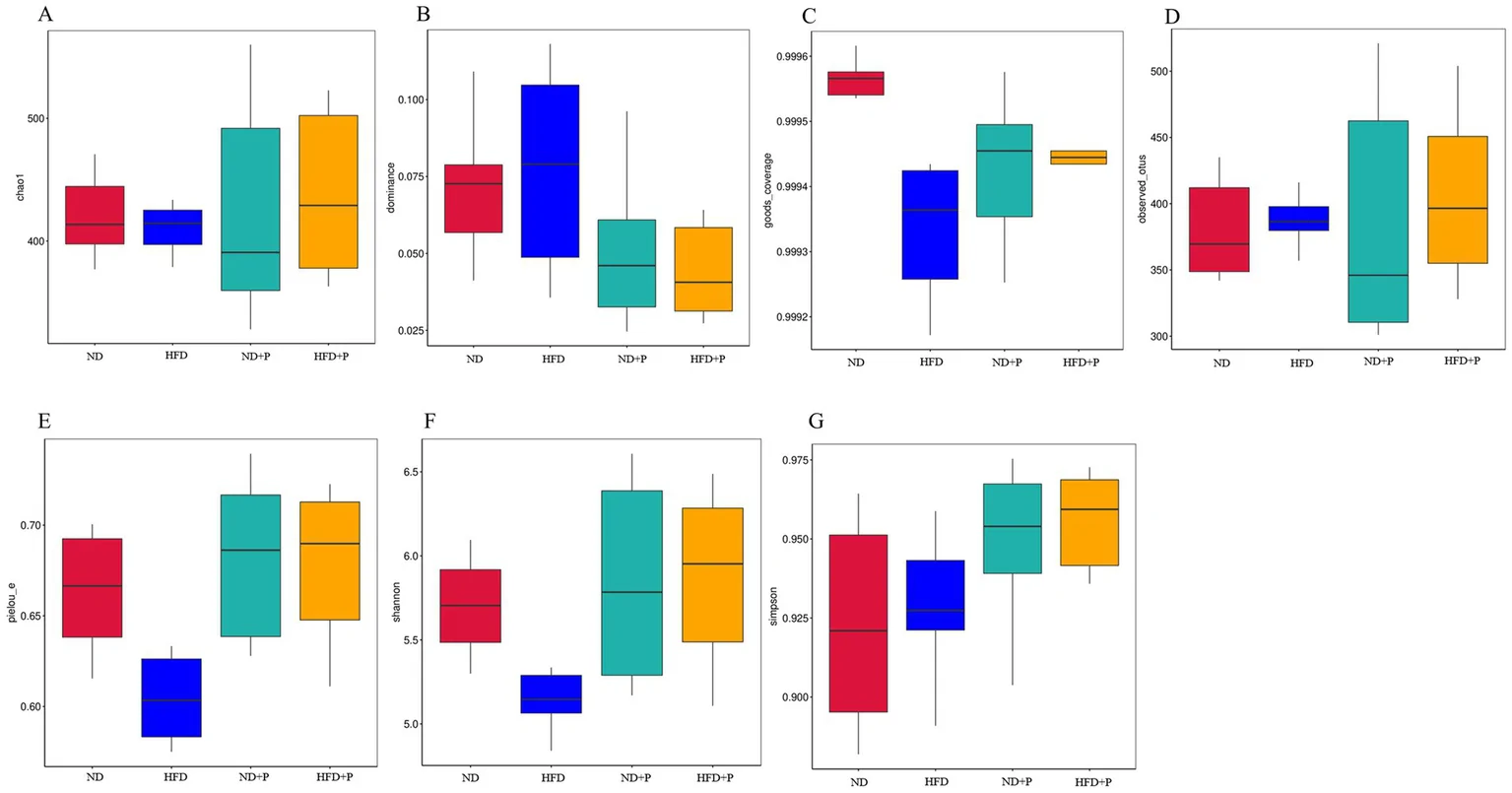
Effects of MCP intervention on the α-diversity indices of fecal microbiota in HFD-fed mice. **(A)** Chao1. **(B)** Dominance. **(C)** Good’s Coverage. **(D)** Observed OTUs. **(E)** Pielou_e. **(F)** Shannon. **(G)** Simpson.

To delve deeper into the alterations in the gut microbiota of mice, we analyzed an analysis comparing the abundances of dominant phyla and genera in each group. Our community composition analysis revealed similarities in the gut microbiota compositions across all groups, with variations in colony relative abundances. The typical gut microbiota comprises mainly Firmicutes and Bacteroidetes, with obesity onset closely linked to the dynamics of these bacterial populations. In Figure 5C, nine phylum categories were identified, including *Bacteroidota, Firmicutes, Verrucomicrobiota*, and *Actinobacterial.* Notably, the HFD group exhibited a significant increase in Firmicutes abundance, whereas MCP intervention restored the relative abundance of Firmicutes and Bacteroidota (Figure 5D). At the family level (Figure 5E), the HFD group exhibited increased abundances of *Clostridiaceae, Erysipelotrichaceae,* and *Peptostreptococcaceae,* while the abundance of *Muribaculaceae* diminished. Following MCP intervention, the abundances of beneficial taxa such as *Bifidobacteriaceae, Akkermansiaceae, Muribaculaceae,* and *Prevotellaceae* were enriched, whereas *Peptostreptococcaceae* declined.

Furthermore, the heatmap in Figure 5F illustrates the distribution of the 35 genera with relatively high abundance. Following MCP intervention promoted the proliferation of beneficial taxa such as *Akkermansia, Lactobacillus, Parabacteroides, Lachnospiraceae_NK4A136_group*, and *Bifidobacterium*, while decreasing the abundance of potentially pathogenic bacteria like *Helicobacter, Mucispirillum*, and *Romboutsia* decreased. Finally, Linear discriminant analysis Effect Size (LEfSe) was utilized to identify key phylotypes (biomarkers, LDA > 4). As depicted in Figure 5G, the discriminative taxa in the HFD + P group comprised *Lactobacillales, Lactobacillaceae, Erysipelotrichales, Erysipelotrichaceae, lleibacterium_valens, lleibacterium*, and Clostridiaceae. In contrast, the core microbiota in the HFD group consisted of *Desulfovibrionaceae, Romboutsia, Lachnospiraceae*, and *Clostridia.*

### 3.7 MCP modifies fecal metabolic profiles

Gut microbiota metabolites act as critical signaling molecules in host metabolic processes. A non-targeted metabolomics analysis of fecal samples was conducted to pinpoint specific metabolic signaling molecules affected by MCP intervention. The number of detected metabolites and quality control in positive and negative ion modes are depicted in Figures 7A,B. A distinct separation of metabolic profiles among different groups is evident in the PCA score plot (Figure 7C). Volcano plot and cluster heatmap analyses identified numerous differential metabolites between the HFD group and the HFD + P group (Figures 7D–H; Supplementary Figure S1). Specifically, in negative ion mode, MCP intervention notably decreased bile acid-related metabolites such as deoxycholic acid, lithocholic acid, and lithocholic acid 3-sulfate (Figure 8). Furthermore, pro-inflammatory and oxidative metabolites including arachidonic acid, prostaglandin H1, prostaglandin E2, and (+/−)9-HpODE were significantly reduced post MCP intervention. The uremic toxin 3-indoxyl sulfate exhibited a significant reduction, while metabolites annotated as 2-Methylbutyric acid and 4-Hydroxybutyric acid showed a significant increase following MCP intervention. Furthermore, levels of antioxidant and anti-inflammatory metabolites, such as ascorbic acid and taurine, known for their anti-inflammatory and barrier-protective properties, were significantly elevated. In positive ion mode (Figure 9), there was a notable decrease in bile acids, specifically taurodeoxycholic acid and taurocholic acid, following MCP intervention. The concentrations of pro-inflammatory compounds, including 5-OxoETE, (+/−)8-HEPE, 13-HPODE, 7-Ketocholesterol, pyroglutamic acid, and hippuric acid, were markedly reduced. Conversely, the secondary bile acid tauroursodeoxycholic acid, along with tryptophan metabolites such as indole-3-acetic acid (IAA), trans-3-indoleacrylic acid, and spermidine, showed significant increases. KEGG enrichment analysis further revealed that these altered metabolites were predominantly associated with bile acid metabolism and tryptophan metabolism (Figures 7H–I). These metabolic shifts offer a biological rationale for the previously documented hepatoprotective effects.

**Figure 7:**
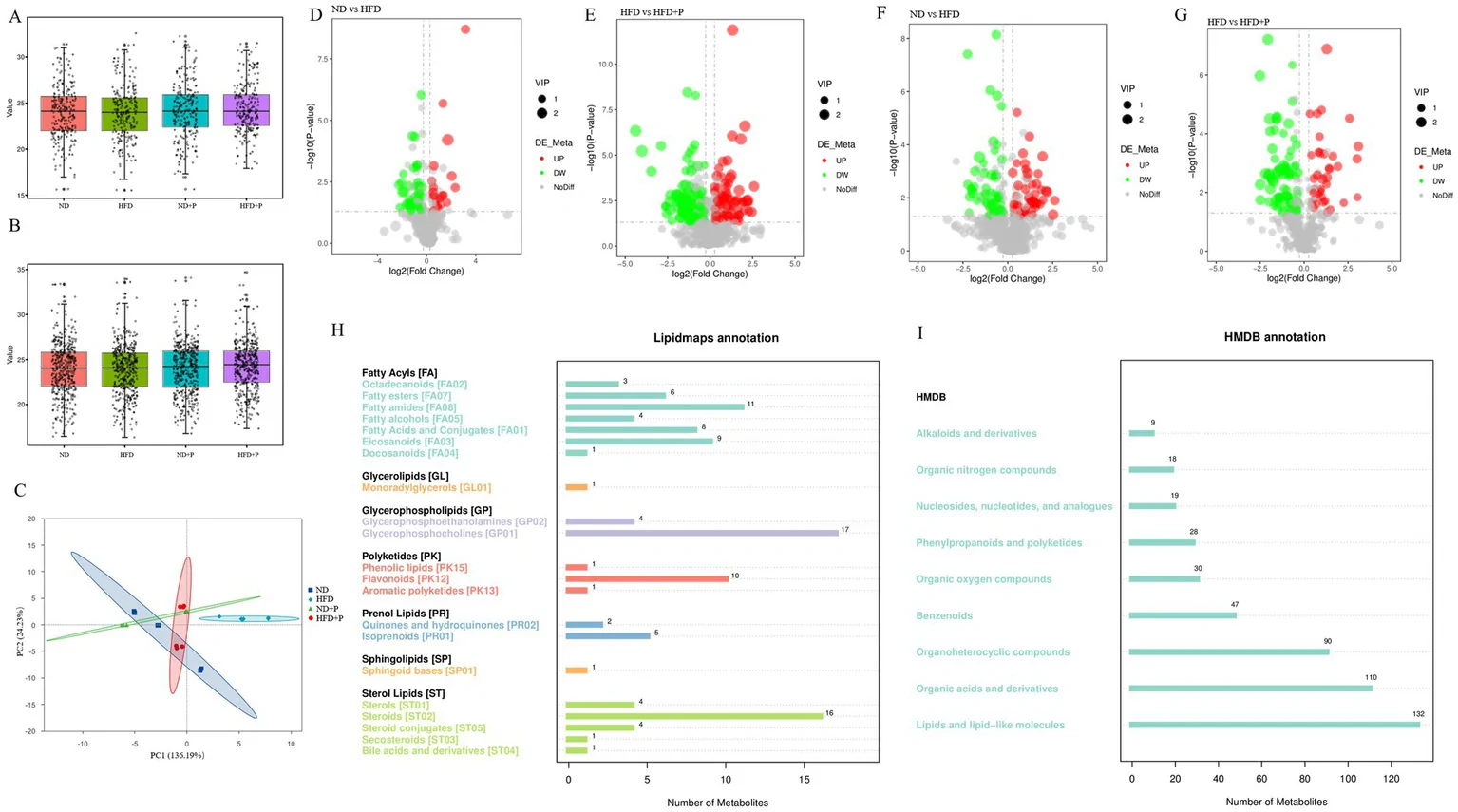
Effects of MCP intervention on fecal metabolites in HFD-fed mice. **(A)** Metabolites detected in negative-ion mode. **(B)** Metabolites detected in positive-ion mode. **(C)** PCA score plot. **(D–G)** Volcano plots of differential metabolites. **(H)** LIPID MAPS classification. **(I)** HMDB classification.

**Figure 8:**
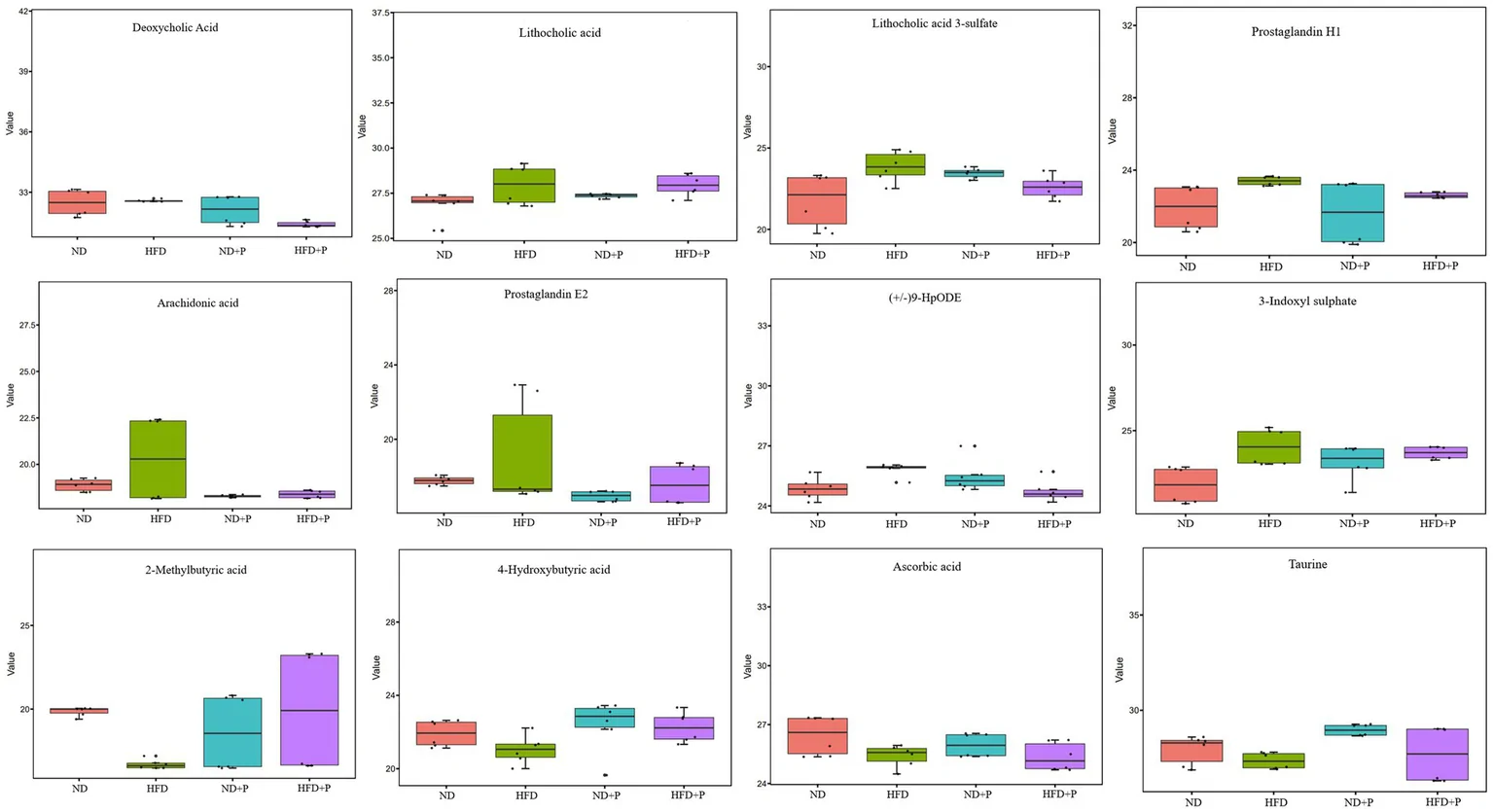
Box plots showing the relative abundance of key differential metabolites detected in negative-ion mode across different groups.

**Figure 9:**
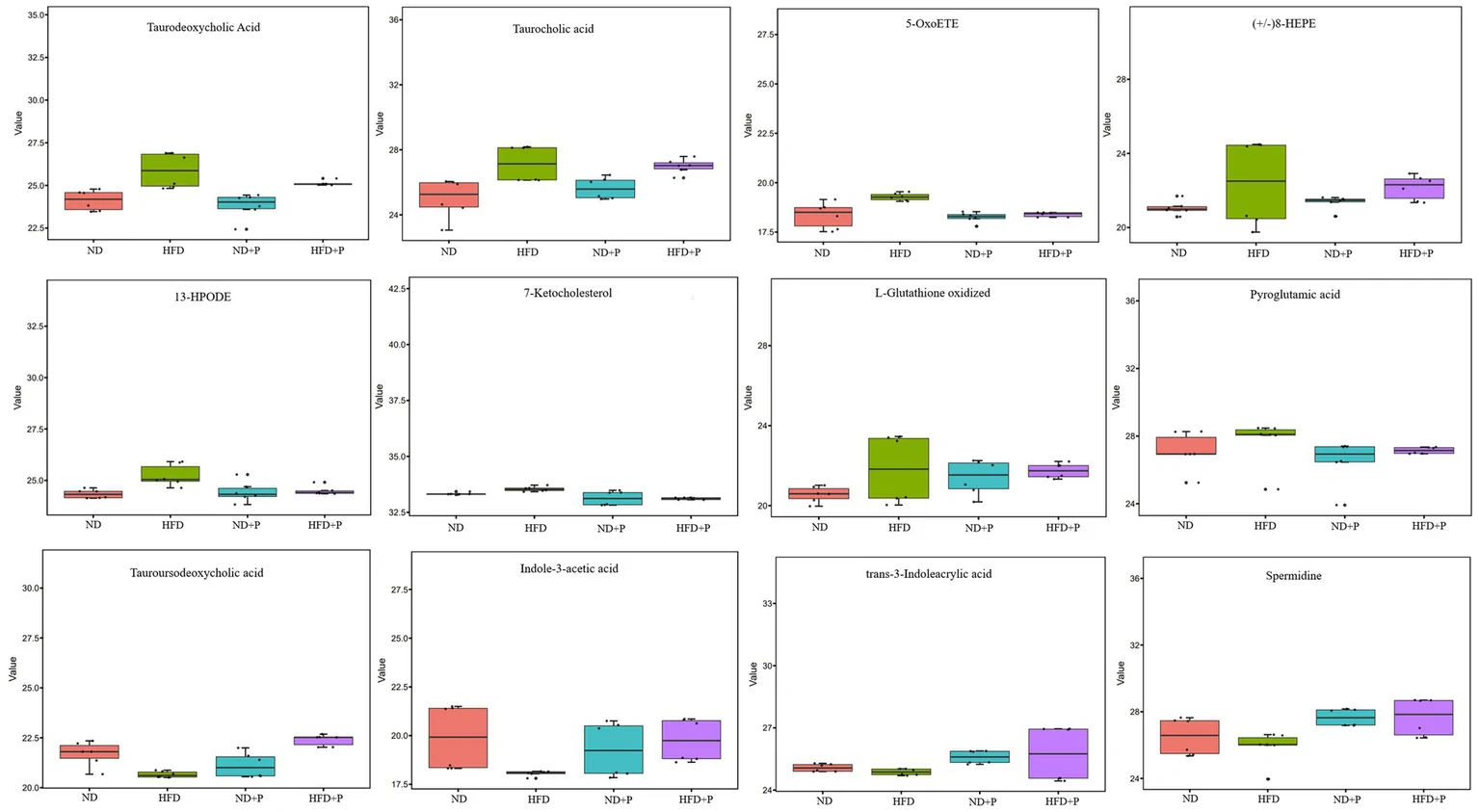
Box plots showing the relative abundance of key differential metabolites detected in positive-ion mode across different groups.

### 3.8 Microbiota-metabolite correlations

A Spearman’s correlation analysis was conducted to assess the potential host-microbiome-metabolite interactions (Figure 10). The findings indicated that *Mucispirillum* exhibited positive correlations with several detrimental metabolites, such as 3-Indoxyl sulphate, Deoxycholic Acid, 5-OxoETE, and Prostaglandin E2. The presence of *Desulfovibrionales* was positively associated with metabolites linked to inflammation and oxidative stress, including (+/−)8-HEPE, 13-HPODE, Arachidonic acid, Prostaglandin E2, and Prostaglandin H1. Conversely, Spermidine showed.

**Figure 10:**
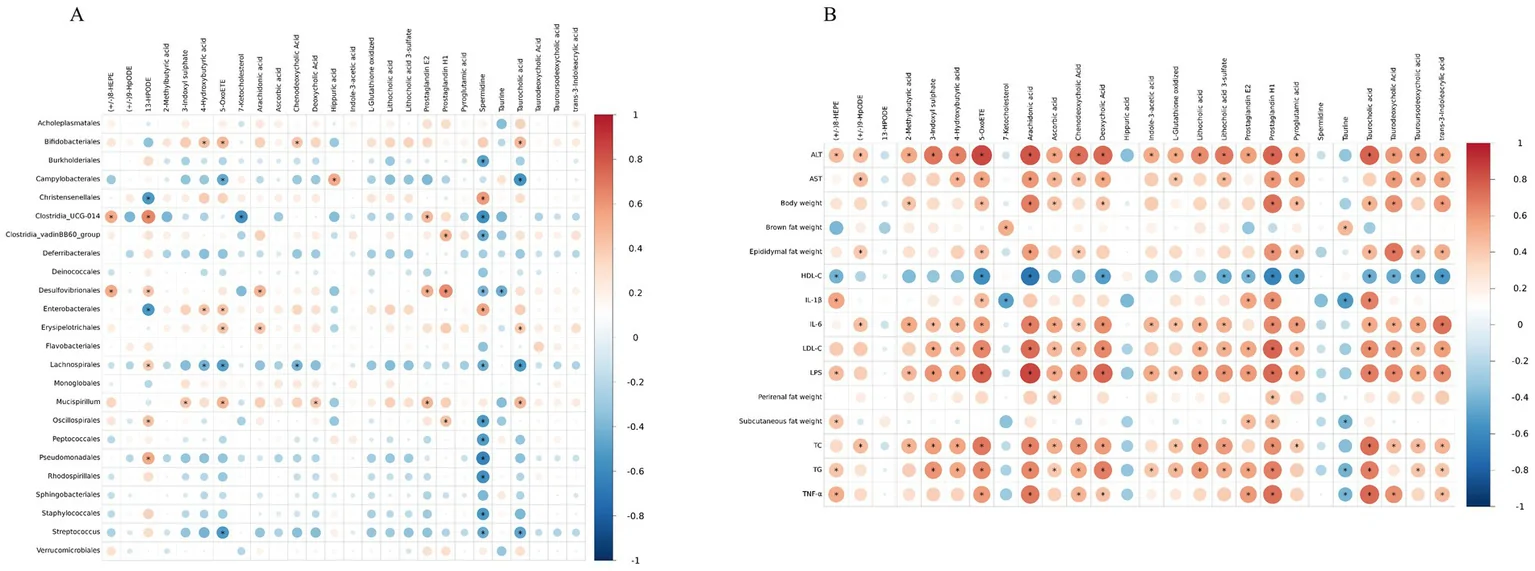
Spearman’s correlation analysis among the gut microbiota, fecal metabolites, and host metabolic parameters in HFD-fed mice. **(A)** Correlation analysis between differential microbiota and metabolites. **(B)** Correlation analysis between differential metabolites and host metabolic parameters.

strong negative correlations with various opportunistic pathogens, including *Burkholderiales, Staphylococcales, Streptococcus, Desulfovibrionales, Oscillospirales,* and *Peptococcales*. Furthermore, the correlation analysis between metabolites and hyperlipidemia-related indicators revealed that ALT and AST exhibited positive correlations with (+/−)9-HpODE, 3-Indoxyl sulphate, Arachidonic acid, 5-OxoETE, Prostaglandin H1, Pyroglutamic acid, and Taurodeoxycholic Acid. Inflammatory markers IL-6 and LPS were positively correlated with 5-OxoETE, Arachidonic acid, Prostaglandin H1, Pyroglutamic acid, and Taurodeoxycholic Acid. Additionally, HDL-C displayed negative correlations with (+/−)8-HEPE, 5-OxoETE, Arachidonic acid, lithocholic acid 3-sulfate, Prostaglandin H1, Prostaglandin E2, and Pyroglutamic acid. Taurine exhibited positive correlations with Brown fat weight and negative correlations with IL-1β, TNF-α, TG, and Subcutaneous fat weight. These correlations suggest potential associations between MCP-induced microbial alterations, metabolic changes, and improvements in HFD-induced metabolic dysfunction.

## 4 Discussion

Hyperlipidemia and related metabolic disorders have become major nutritional and public health concerns worldwide (28). Dietary peptides derived from milk proteins have attracted increasing attention as functional food ingredients; however, whether peptide preparations retain metabolic benefits after gastrointestinal processing remains insufficiently understood. In the present study, we investigated the metabolic effects of Alcalase-hydrolyzed casein peptides after simulated gastrointestinal digestion (MCPs) in HFD-fed mice. Our findings demonstrate that MCP supplementation attenuated body weight gain and hepatic steatosis, while modulating gut microbiota composition and fecal metabolic profiles. These alterations were accompanied by reduced LPS levels, improved intestinal inflammatory status, and the regulation of hepatic lipid metabolism and oxidative stress, suggesting a potential role of the gut microbiota-liver axis in MCP-mediated metabolic improvement.

The physiological manifestations of hyperlipidemia typically originate from the dysregulation of energy homeostasis, culminating in the pathological expansion of adipose tissue and subsequent ectopic lipid deposition (29). Upon the consumption of a high-fat diet, the growth curve of mice in the HFD group diverged progressively, resulting in substantial body weight gain and fat mass accumulation. Following MCP supplementation, body weight gain and white fat pad accumulation were significantly attenuated, aligning with previous studies reporting the anti-obesity effects of milk-derived peptides (30). Adipose tissue is not merely an energy reservoir (31, 32). In obesity, hypertrophic adipocytes undergo enhanced lipolysis and release excessive FFAs into circulation (33). Due to the anatomical proximity and vascular linkage via the portal vein, the liver acts as a major metabolic organ responsible for processing adipose-derived FFAs and gut-derived nutrients (34). Our histopathological analyses visually confirmed that HFD feeding induced marked hepatic lipid accumulation and hepatocyte swelling. The resulting lipotoxicity may compromise hepatocyte integrity, evidenced by the significant leakage of intracellular transaminases (ALT and AST) into the bloodstream (35). Notably, MCP intervention was associated with a reduction in adipocyte enlargement, reduced liver weight, and normalized serum transaminase levels. Together, these observations indicate that MCPs may alleviate the metabolic burden imposed by HFD by improving systemic lipid handling.

The progression from hepatic steatosis toward metabolic dysfunction is closely associated with local and systemic chronic inflammation, which may be influenced by intestinal barrier disturbances (11, 36). The intestinal epithelium serves as a critical interface limiting the systemic entry of luminal antigens and pathogenic endotoxins. Pathological examination in our study indicated that HFD consumption induced colonic mucosal damage, crypt destruction, and extensive inflammatory cell infiltration. This intestinal morphological impairment and inflammatory activation likely facilitated the translocation of lipopolysaccharide (LPS) into the circulation. Upon reaching the liver, LPS binds to Toll-like receptor 4 (TLR4) on Kupffer cells and hepatocytes, triggering subsequent inflammatory responses (37). Consequently, we observed markedly elevated serum levels of pro-inflammatory cytokines (TNF-α, IL-1β, and IL-6) and LPS in the HFD group. Following MCP supplementation, colonic morphology was improved, accompanied by reduced inflammatory responses and circulating LPS levels. While direct markers of physical barrier permeability were not evaluated in the present study, these histopathological and biochemical improvements suggest that the anti-inflammatory effects of MCP are closely linked to the improvement of intestinal homeostasis.

The integrity of the gut barrier and host metabolic health are profoundly modulated by the dynamic composition of the gut microbiota (32). Prolonged HFD consumption invariably induces microbial dysbiosis, often accompanied by alterations in the relative abundance of Firmicutes and Bacteroidetes, which is positively correlated with enhanced dietary energy harvest and exacerbated fat storage (38). Our data revealed that MCP altered the relative abundance of major bacterial taxa associated with metabolic health. Beyond macro-level compositional shifts, the specific enrichment of health-associated bacterial taxa provides critical insights. At the genus level, *Bifidobacterium*, *Akkermansia*, and *Lactobacillus* exhibited significant upward trends following MCP treatment. While our study solely observed compositional shifts, previous independent research has robustly associated *Bifidobacterium* has been associated with improved intestinal barrier function in previous studies, thereby preventing LPS translocation (39). Similarly, *Akkermansia* and *Lactobacillus* have been associated with improved intestinal metabolic homeostasis in previous studies (40, 41). Conversely, our LEfSe and abundance analyses revealed that HFD feeding enriched detrimental taxa such as *Desulfovibrionaceae* and *Peptostreptococcaceae*. According to previous reports, *Desulfovibrionaceae* are known sulfate-reducing bacteria that produce hydrogen sulfide (H_2_S), a genotoxic compound that damages the intestinal epithelium and provokes mucosal inflammation (42, 43). MCP supplementation may create a microbial environment favorable for health-associated taxa while limiting the expansion of opportunistic microorganisms under HFD conditions. However, it is crucial to acknowledge that our 16S rRNA sequencing data primarily reflect taxonomic compositional shifts rather than direct functional, these specific microbiome-host interactions warrant targeted experimental verification in future studies.

Microbial-derived metabolites act as crucial signaling molecules linking the gut microbiota to host metabolic phenotypes (44). Our non-targeted metabolomics and Spearman’s correlation analyses provide compelling insights into the correlations between MCP-induced microbial shifts and physiological improvements. A potential mechanism associated with these benefits involves the modulation of the bile acid (BA) pool. The liver synthesizes primary bile acids, which are subsequently deconjugated and biotransformed into secondary bile acids by gut microbiota containing bile salt hydrolase (BSH) activity, such as *Bifidobacterium* and *Lactobacillus* (45). In our study, MCP intervention significantly reduced features annotated as fecal bile acid-related metabolites, including deoxycholic acid and lithocholic acid, as well as taurodeoxycholic acid (TDCA), while increasing a metabolite feature annotated as tauroursodeoxycholic acid (TUDCA). Notably, the signal assigned to TDCA exhibited significant positive correlations with liver injury markers (AST, ALT) and LPS. In contrast, TUDCA has been reported to regulate FXR and TGR5 signaling pathways involved in hepatic lipid metabolism (46, 47). Beyond bile acid remodeling, our metabolomics profiling revealed a significant enrichment of features putatively identified as tryptophan metabolites, specifically indole-3-acetic acid (IAA) and trans-3-indoleacrylic acid, in the MCP-treated mice. These indole derivatives can be generated through gut microbiota-mediated metabolism of dietary tryptophan (48). IAA is a well-documented endogenous ligand for the Aryl Hydrocarbon Receptor (AhR) (49). These metabolites have been reported to participate in intestinal immune regulation through AhR-related pathways, which may provide a possible explanation for the improved colonic phenotype observed after MCP supplementation. Additionally, levels of spermidine, a polyamine closely associated with autophagy induction and potent anti-inflammatory effects, were significantly elevated post-intervention and negatively correlated with pathogens like *Desulfovibrionales* (50). Concurrently, MCP intervention markedly ameliorated systemic lipid peroxidation and eicosanoid-driven inflammation. HFD feeding typically overactivates the cyclooxygenase (COX) and lipoxygenase (LOX) pathways, leading to the excessive production of pro-inflammatory lipid mediators. We observed that metabolites annotated as arachidonic acid and its downstream inflammatory derivatives, such as prostaglandin E2 (PGE2), prostaglandin H1, and 5-OxoETE, were significantly depleted following MCP treatment. PGE2 is a potent vasodilator and immune cell chemoattractant that exacerbates local tissue inflammation (51). The concurrent reduction of 3-indoxyl sulfate and oxidized lipids such as (+/−)9-HpODE further suggests that MCP supplementation was associated with reduced oxidative and inflammatory metabolic signatures induced by HFD feeding. Conversely, endogenous antioxidants such as ascorbic acid and taurine were significantly elevated. Taurine, in particular, exhibited positive correlations with brown fat weight and negative correlations with TG and TNF-α. Taurine not only conjugates with bile acids to improve their solubility and excretion but also directly scavenges reactive oxygen species (ROS) in hepatocytes (52, 53). Collectively, these metabolic alterations indicate a shift from a pro-inflammatory state toward a more balanced metabolic profile, which may contribute to the protective effects of MCPs. Nevertheless, a notable limitation of the current study is that these metabolic insights are derived from untargeted metabolomics. Because targeted bile acid analysis was not performed to provide absolute quantification and rigorous structural validation, the TUDCA-related metabolic changes should be interpreted cautiously.

Furthermore, these intestinal and metabolic improvements parallel the modulation of hepatic molecular pathways. HFD feeding significantly upregulated the expression of sterol regulatory element-binding protein 1c (SREBP-1c) in the liver. As the master transcriptional regulator of lipogenesis, hyperactivated SREBP-1c directly stimulates the expression of acetyl-CoA carboxylase (ACC) and glycerol-3-phosphate acyltransferase 1 (GPAT1), the rate-limiting enzymes responsible for *de novo* fatty acid and triglyceride synthesis, respectively (54). The significant downregulation of SREBP-1c, ACC, and GPAT1 following MCP intervention may be associated with alterations in the gut microbiota-liver axis. Specifically, the reduction in circulating LPS levels may contribute to the attenuation of inflammatory signaling, while the changes in microbial-derived metabolites may participate in the regulation of SREBP-1c transcription via FXR and AMP-activated protein kinase (AMPK) pathways (55, 56). Given that pro-inflammatory cytokines and chronic endotoxemia can exacerbate hepatic steatosis by directly stimulating SREBP-1c expression and *de novo* lipogenesis, the reduction in LPS serves as a crucial link between improved colonic health and restored hepatic lipid metabolism. Concurrently, severe metabolic overload typically triggers hepatic oxidative damage (57). Our results demonstrated that MCP increased hepatic Nrf2 and HO-1 expression. The enhanced Nrf2/HO-1 response may be associated with improved microbial-metabolic profiles, including the increased abundance of beneficial taxa and elevated levels of antioxidant-related metabolites (58). These integrated molecular findings support the involvement of MCP in attenuating hyperlipidemia through the coordinated regulation of intestinal and hepatic metabolic responses.

In summary, this study provides comprehensive evidence that MCP is an effective dietary intervention for ameliorating HFD-induced hyperlipidemia and its associated metabolic morbidities. The primary conclusion of our research is that MCP exerts its metabolic benefits through a highly coordinated gut microbiota-metabolite-liver axis. MCP significantly attenuates body weight gain, prevents adipocyte hypertrophy, and alleviates hepatic steatosis and hepatocyte injury. Underlying these systemic improvements, MCP improves colonic morphology and effectively remodels the gut microbiota, specifically reducing pathogenic *Desulfovibrionaceae* while enriching health-associated taxa such as *Bifidobacterium, Akkermansia,* and *Lactobacillus.* Critically, this microbial restructuring translates into an optimized metabolic milieu. MCP intervention significantly elevates barrier-protective tryptophan metabolites (IAA) and hepatoprotective secondary bile acids (TUDCA), while concurrently reducing circulating endotoxins (LPS), cytotoxic bile acids, and pro-inflammatory eicosanoids (arachidonic acid, PGE2). Consequently, this fortified gut-barrier and beneficial metabolic signaling synergize to alleviate hepatic inflammatory stress, leading to the targeted suppression of *de novo* lipogenesis (SREBP-1c/ACC/GPAT1 pathway) and the activation of antioxidant defenses (Nrf2/HO-1 pathway). Importantly, this study highlights that casein peptides obtained after enzymatic hydrolysis and simulated gastrointestinal digestion retain metabolic regulatory potential, supporting their further exploration as dairy-derived functional ingredients.

## Data Availability

The raw 16S rRNA sequencing data generated in this study have been deposited in the NCBI Sequence Read Archive (SRA) under the BioProject accession number PRJNA1520775. All data are available from the corresponding authors upon request.
